# An umbrella review synthesising published systematic review evidence for self-harm and suicidality among patients diagnosed with an eating or feeding disorder

**DOI:** 10.1186/s40359-026-04609-z

**Published:** 2026-05-26

**Authors:** Anoushka Baluja, Roger T. Webb

**Affiliations:** 1 School of Health Sciences, The University of Manchester, Stopford Building, Oxford Road, Manchester, M13 9PL UK; 2 Division of Psychology & Mental Health, School of Health Sciences, The University of Manchester, Jean McFarlane Building, Oxford Road, Manchester, M13 9PY UK

**Keywords:** Feeding and eating disorders, Anorexia nervosa, Bulimia nervosa, Self-harm, Suicidality, Suicidal ideation, Suicide

## Abstract

In recent years there has been a proliferation of systematic reviews on the association between feeding and eating disorders and self-harm and suicidality. This study aims to conduct an umbrella review, providing a top-level synthesis of this information. A systematic search was performed using two databases (MEDLINE and APA PsycInfo), with inclusion criteria of studies identifying as systemic reviews, written in English, and researching the association between feeding and eating disorders and self-harm and/or suicidality. The Joanna Briggs Institute (JBI) Critical Appraisal tools for JBI systematic reviews was used to assess the quality of the included studies. A total of 14 systematic reviews and meta-analyses, 10 systematic reviews accompanied by meta-analyses (4 standalone systematic reviews and 10 systematic reviews and meta-analyses) were included in the umbrella review. A strong prevalence between non-suicidal self-injury, suicidal ideation and suicide attempts was found, emphasising the importance of sufficient self-harm and suicidality screening measures for patients with feeding and eating disorders. The binging-purging subtype of anorexia nervosa was identified as independently associated with suicide attempts, and higher levels of impulsivity. The integrated motivational-volitional model can be used as a basis for comparing the progression to suicidality in patients with and without feeding and eating disorders, allowing more clarity on if and how this differs. This review found the onset of feeding and eating disorder and self-harm and suicidal behaviours can vary between the individual and suggests this may be due to different reasons for engaging in a feeding and eating disorder, self-harm, and suicidal behaviours specific to the individual. The interplay between feeding and eating disorders and self-harm and suicidality remains largely unclear, and further longitudinal research within this area is needed.

## Background

### Feeding and eating disorders

Feeding and eating disorders are defined in the ICD-11 as “abnormal eating and feeding behaviours that are not better accounted for by other health conditions and are neither developmentally appropriate nor culturally sanctioned.” [1] Excessive weight loss, present in some feeding and eating disorder, can lead to secondary endocrine and metabolic disturbances [2, 3] resulting in significant physical complications across multiple organ systems [4]. Feeding and eating disorders are becoming increasing prevalent, with a lifetime prevalence of 3.35–18.6% for women and 0.8%—6.5% for men [5]. Both the International Classification of Disease 11 (ICD 11) and Diagnostic and Statistical Manual of Mental Disorders V (DSM V) recognise different types of feeding and eating disorders: anorexia nervosa (AN), bulimia nervosa (BN), Overeating or Binge Eating disorder (BED) and other unspecified eating disorders (OSFED) [6]. The ICD 11 also include three more categories, Avoidant-Restrictive Food Intake Disorder (ARFID), Pica, and Rumination-Regurgitation Disorder, which are commonly associated with children (Table 1) [7].

**Table 1:** The key diagnostic features of the different types of feeding and eating disorders

	Anorexia Nervosa	Bulimia Nervosa	Binge Eating Disorder	Avoidant Restrictive Intake Disorder	Pica	Rumination-regurgitation disorder
Eating	Restrictive	Irregular patterns, can include some restriction	Irregular	Restrictive of all or selective foods	Includes non-food objects (e.g. dirt, paper) or large quantities of raw food ingredients (e.g. salt, corn flour)	Intentional and repeated swallowing of food back into the mouth
Binging	Can be present	Regular, and with compensation	Regular, and without compensation	Not present	Not present	Not present
Purging, overexercise, or weight control behaviours	Can be present	Regular as compensatory behaviours	Not regular	Not present	Not present	Not present
Typical weight	Below expected range	Above or within expected range	Above or within expected range	Below expected range, with nutritional deficiency	Unintentional weight loss and nutritional deficiency can occur	Not affected by the condition
Body image	Overvaluation	Overvaluation	Overvaluation may be present	No overvaluation	No overvaluation	No overvaluation

Adapted and expanded from: Hay P. Current Approach to Eating disorders: a Clinical Update. J. Intern. Med [Internet]. 2020 Jan 14;50(1):24–9. Available from: https://www.ncbi.nlm.nih.gov/pmc/articles/PMC7003934/

The DSM V designates two subtypes of anorexia nervosa: the restricting subtype (AN-R), characterised by an absence of regular binge eating or purging three months prior to the diagnoses and the binging-purging subtype (AN-BP), characterised by binge eating, purging (e.g. self-induced vomiting, misuse of laxatives) or a combination of both behaviours three months prior to the diagnosis [8]. In AN-BP, binging and/or purging behaviours may occur, however both subtypes of anorexia nervosa are conditions characterised by self-starvation, and being underweight is a diagnostic requirement. In bulimia nervosa, cycles of binging and purging are the predominant feature, occurring regularly, and patients may be within or above the weigh recommendations for the sex, age, and height. Consistent with overvaluation of weight and size and body image is of paramount importance to self-view for patients with anorexia or bulimia nervosa [9]. Notably, feeding and eating disorder types and subtypes are not fixed and patients can migrate from one form of an feeding and eating disorder to another [10]. Behaviours that are similar to those found in eating disorders but occur at a lower frequency or intensity and do not meet diagnostic requirements for a feeding and eating disorder, are termed as disordered eating behaviours [11].

### Self-harm and suicidality

Self-harm is defined as an expression of emotional distress through purposeful and intentional self-poisoning or self-injuring behaviours, irrespective of the motivation or purpose of the act [12]. Suicidality consists of behaviours with suicidal intent, ranging from suicidal ideation (thinking or formulating plans for suicide), suicide attempts and completed suicide. A range of factors are associated with self-harm and suicide, including biological, mental health related problems, body mass index, emotional regulation and adverse childhood experiences [13]. There is a higher prevalence of self-harm and suicidality in patients with eating disorders [14–16]. Feeding and eating disorders are also associated with increased mortality rates [12], and suicide makes a large contribution towards this [17]. Anorexia nervosa, specifically, has the highest mortality rate of any psychiatric disorder, with approximately 5% of patients dying within four years of the diagnosis [18]. Suicide is a significant cause of mortality in patients with anorexia nervosa, with one meta-analysis concluding 20% of the individuals with fatal anorexia committed suicide [19]. Other causes of mortality in patients with anorexia nervosa result from physical complications of the disease across a range of organ systems, with particular associations with pneumonia and diabetes [18].

Significant research has been done to understand why suicide occurs. Multiple theories and frameworks have been proposed, aiming to provide an explanation of the factors that lead up to suicidal thoughts and behaviours. These frameworks approach the subject from a range of perspectives and generally can be categorised into using a sociological, psychological, sociocultural, developmental, or integrated psychological lens [20], which focuses on the progression of suicidal ideation to action. One of the major ideation-to-action theories is the integrated motivational-volitional model of suicidal behaviour (IMV model) [21].

This framework was chosen over alternative theories as it conceptualises suicide with a biological, social, and psychological influences. This offers a more comprehensive account of suicide and better reflects the complexity of patient presentations within clinical practice.

While other ideation-to-action models consider the development of suicidal ideation to behaviour, the IMV model presents a more detailed categorisation of moderators involved, separating them into pre-motivational, motivation and volitional phases. Unlike other ideation-to-action models, it explicitly distinguishes the development of suicidal ideation from factors that facilitate suicidal behaviour. In terms of clinical practice, this is particularly useful when considering suicide risk in patients with feeding and eating disorders and strategies for preventing or minimising the risk of suicidal behaviour.

This review aims to assess its results in conjunction with this model, using it as a framework to identify and structure its findings.

### The integrated motivational-volitional model of suicidal behaviour (O’Conner 2018) [22]

The integrated motivational-volitional model of suicidal behaviour (IMV) has combined multiple previous theories to provide a biopsychosocial framework that describes the pathway to suicidal ideation and behaviour. It is a second-generational model consisting of three phases.

**Fig. 1:**
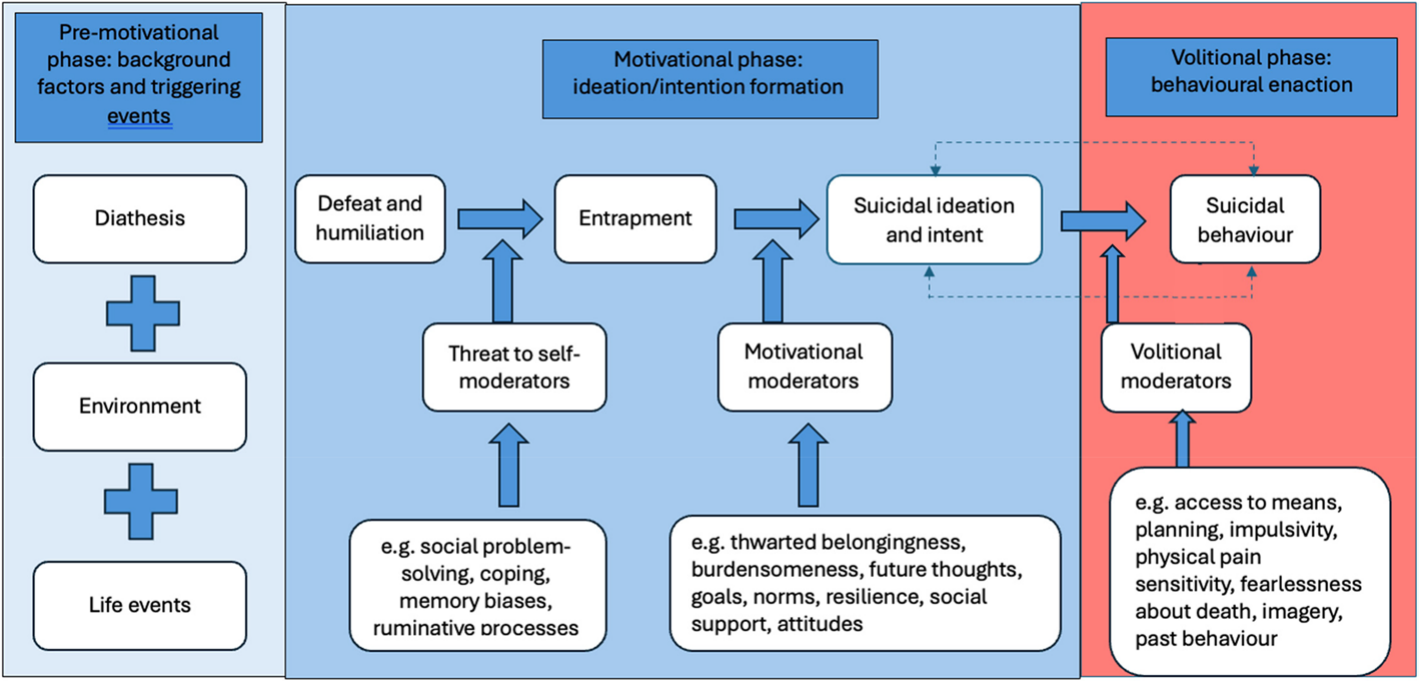
A flowchart depicting the IMV model [19]. Image recreated from Fig. 1, O’Connor RC, Kirtley OJ. The integrated motivational–volitional model of suicidal behaviour. Philos Trans R Soc Lond B Biol Sci Sciences [Internet]. 2018 Jul 16;373(1754):20170268. Available from: 10.1098/rstb.2017.0268

The first phase, the pre-motivational phase, consists of the ‘diathesis-environment-life’ triad. Diathesis consists of biological, genetic, or cognitive factors or characteristics that increases risk of suicide. The second phase, the motivational phase, consists of psychological processes that result in suicidal ideation. This includes defeat, humiliation with no perceived escape, progressing to a sense of entrapment and then suicidal ideation. Additional factors that influence these core feelings and are depicted in Fig. 1 below. The final phase is the Volitional phase, which depicts the pathway for suicidal ideation to attempting suicide/suicide. The volitional moderators that govern this transition, include acquired capability, as well as other environmental, psychological, social, or physiological factors (e.g. impulsivity, physical pain sensitivity, fearlessness about death). The pre-motivational factors are thought to increase the risk of suicide by influencing the constructs within the motivational and volitional phases. The threat to self, motivational and volitional moderators found in these phases contribute directly to the development of suicidal ideation and behaviour within an individual. This process can be visualised in Fig. 1.

There has been a proliferation of research done on the association between feeding and eating disorders and self-harm and suicide in recent years. In the current state of research, no previous umbrella reviews studying feeding and eating disorders and self-harm, or suicidality have been conducted. An umbrella review by Tan et al. [23] studied the association between eating disorder and mental health and found that self-harm is a significant correlate of feeding and eating disorders amongst adolescents and adults. Patients with feeding and eating disorders were also found to be at higher risk of suicide, however the study concluded further research on the risk factors for suicide was needed. A conference review paper by Juli et al. established a significant prevalence of self-harm in patients with eating disorders and is associated with more severe psychopathology [24]. This increases the patient’s risk of more severe depressive and anxiety symptoms and suicide attempts. Furthermore, evidence supporting a significant association between suicidal ideation and behaviour in patients with feeding and eating disorders was synthesised in a narrative review by Smith et al. [25], however there was limited research available. This was especially apparent for longitudinal studies, resulting in the timing of the risk for dying form suicide remaining unclear. This umbrella review aims to synthesise information from systematic reviews, with the purpose being to provide a better understanding of the relationship between feeding and eating disorders and self-harm and suicidality. It also aims to use the IMV as an interpretational framework.

## Method

PROSPERO [26], an international database that was created by the University of York, provides the first global, online facility to register and review the aims of systematic reviews researching health and social care. PROPSPERO registration of this review was attempted, but due to recent restrictions on student projects and the required study design (intervention review only) due to recent significantly increasing demand, this was not possible.

### Ethical considerations

Ethical approval from the University of Manchester Research Committee was not needed for this project.

### Search strategies and databases

In consultation with an experienced librarian, a thorough and comprehensive literature search was conducted, looking at all articles that had been published until the search date: 15th May 2025. The search was conducted on the OVID platform on two electronic databases: MEDLINE and APA PsycInfo. The inclusion criteria were (1) identified and reported as a systematic review by the author(s); and (2) including and reporting on feeding and eating disorders and self-harm and death by suicide, (3) written in English. Articles that were not systematic reviews, e.g. narrative review, or meta-analysis that did not conduct a systematic review of the literature. The following search terms to cover suicidality and self harm were used: Suicid*, selfharm, self harm, NSSH, selfinjur*, self injur*, NSSI, self poison*, self mutilat*, selfcut, self cut, parasuicide*, para suicid*. The following terms for feeding and eating disorders were used: 'eating disorder', 'disordered eating', anorexi*, bulimi*, 'binge eat*', purg*, 'eating patholog*', AN-BP, AN-R, BN, BED, EDNOS, OSFED, (eating disorder not specifi*), (other specified feeding or eating disorder). Boolean operators (or/and) were used to ensure studies were related to both topics. The search terms were limited to appearing in the title or abstract, as it was decided that articles where this did not happen, would not be sufficiently relevant.

The decision to include eating disorders and well as disordered eating within the search terms was discussed thoroughly between the two authors. While a diagnosis of an eating disorders require diagnostic criteria and disordered eating does not, there are many overlapping features between them. It is arguable that diagnostic criteria serve a more pragmatic function in determining who does and does not qualify for further intervention in health systems that have limited resources. Some individuals with disordered eating may go on to be diagnosed with an eating disorder imminently or at some later time. While results will detail separately results regarding eating disorders and disordered eating, it was decided the data gathered from research about eating disorders and disordered eating would be beneficial for the umbrella review.

All studies from the database search results underwent full-text screening and the search term ‘systematic review’ applied to the results to ensure that the study had been reported as a systematic review by one author (AB), as per the inclusion criteria. The following data was extracted, by the same author, from studies that met the inclusion criteria: (1) citation details; (2) objective of the systematic review, (3) number of included primary studies; (4) type of feeding and eating disorder or transdiagnostic process being studied; (5) type of self-harm or suicidality or risk factors for self-harm or suicidality; (6) population description given; (7) presence of meta-analysis component and its findings; (8) outcomes and findings. For any studies where the relevance of the paper was unclear, they were discussed with author RW and an agreement was made, with respect to the inclusion criteria.

### Quality assessment

The quality and risk of bias of the included studies were assessed using the Joanna Briggs Institute checklist for Systematic Reviews and Review Syntheses independently by author AB and RW [27]. The checklist consists of 11 questions, each with the option of with Yes, No, Unclear, or Not Applicable as answers, with the final score ranging from 0–11. An additional review was done independently by RW, and this table can be viewed in the appendix.

The JBI allows the methodological quality of the studies to be assessed, determining the extent of bias in the design, conduct and analysis. Additionally, common deficiencies found in systematic reviews can be identified and highlighted as an area of caution for future research. However, it is important to acknowledge the JBI tool is a relatively crude way of measuring bias, relying on subjective ‘Yes’, ‘No’, or ‘Unsure’ answers. For those who do not have extensive experience in research statistics, accurately determining whether the method for combining studies was appropriate may be challenging, possible resulting in errors in judgment when reviewing the study due to lack of knowledge on the topic. Due to the time constraints of this project, reviewers had to rely on the information available in the published report, as contacting the author and clarifying areas of uncertainty was not feasible.

Risk of bias was assessed using the risk of bias in systematic reviews (ROBIS) [28] tool was additionally carried out by the author AB. This tool comprises of three phases. Phase 1 assesses relevance. Phase 2 is focuses on bias in the review process and is comprised of 4 domains: (1) study eligibility criteria, (2) identification and selection of studies, (3) data collection and study appraisal, (4) synthesis and finding. Signalling questions, 5 in the domain 1–3 and 6 in domain 4, are used to determine the concern for risk of bias for that section. Each signalling questions can be answered with yes, probably yes, probably no or not indicated, which then allows an overall level of concerns to be determined for that domain (low, high, or unclear). A global risk of bias for the paper is then determined for the paper in phase 3.

ROBIS enables risk of bias in a previously conducted systematic review to be assessed, focusing on different potential sources of bias. Similar, to the JBI, it also identifies common limitations across systematic reviews. By identifying reviews with a higher risk of bias, it indicates which results should be interpreted with caution. However, also congruent with the JBI, answers are subjective and are also determined in a relatively crude manner with answer options of ‘Yes’, ‘Probably yes’, ‘Probably no’, ‘No’, or ‘Not indicated’.

More robust tools to review bias, such as A MeaSurement Tool to Assess systematic Reviews 2 (AMSTAR 2), were considered, however ultimately it was decided that this would not be suitable. AMSTAR 2 is typically used for randomised control trial studies and therefore is not applicable to the more narrative style of this umbrella review. AMSTAR 2 typically assesses systematic reviews focusing on interventions, therefore would not be suitable to the more descriptive synthesis of information found in this review [29].

## Results

**Fig. 2:**
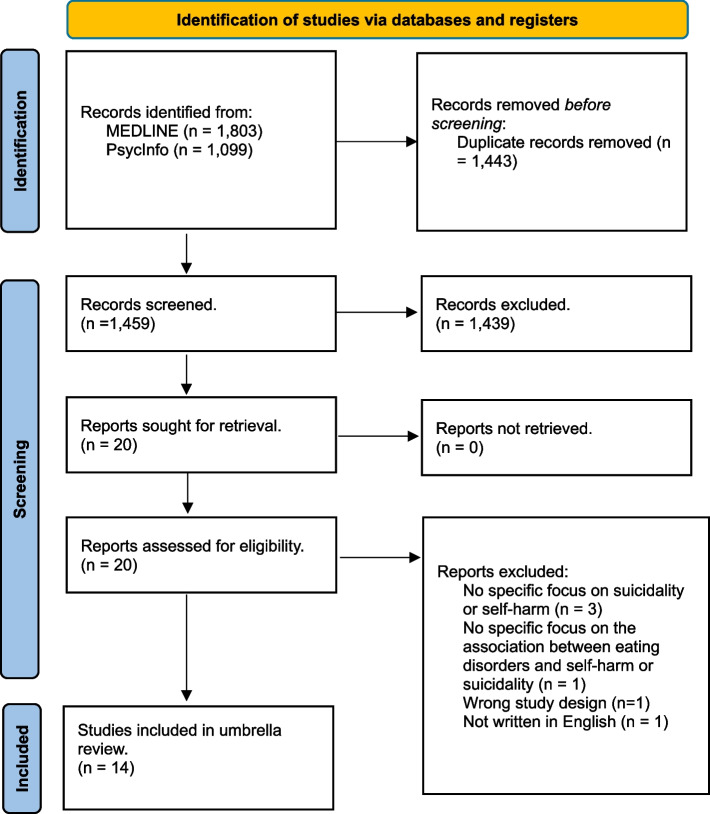
PRISMA flow diagram for the initial search of new systematic reviews which included searches of databases and registers only. Source: Page MJ, et al. BMJ 2021;372:n71. 10.1136/bmj.n71. This work is licensed under CC BY 4.0. To view a copy of this license, visit

Upon running the search for self-harm or suicidality, 70,196 studies were identified. When running the search for feeding and eating disorders, 75,702 studies were identified. Out of these studies, 1,459 were identified as containing terms in the title or abstract relevant to both self-harm or suicidality and feeding and eating disorders. Once searching for the term systematic review or meta-analysis within these studies, 20 studies remained. No studies were removed after title and abstract screening. Upon full text screening, 6 studies were removed, leaving 14 studies which met the inclusion criteria and were included in the review. The reasons for excluding 6 studies after full text screening are described in Fig. 2.

A second search was carried out, across the same 2 databases, to specifically check for any literature with the new categories of feeding and eating disorders in the ICD 11. The same search terms used in the original search for self-harm, suicidality and systematic reviews were used and the terms ‘Pica’ OR “ARFID’ OR “Avoidant restrictive food intake disorder’ OR ‘rumination-regurgitation disorder’ was included. 6 initial results were found, however after full-text abstract screening all the studies were excluded. 5 studies were excluded due to no specific focus on suicidality or self-harm and 1 due to no specific association on the relationship between feeding and eating disorders or suicidality.

### Characteristics of included studies

The extracted data from the 14 included systematic reviews and meta-analysis (published between 2004 and 2025) are included in Table 2. Out of the 14 articles, 10 included meta-analyses. 7 studies looked at the prevalence of NSSI and suicidality among feeding and eating disorders, and 1 study examining the prevalence of NSSI in disordered eating. Two of these studies, specifically focused on adolescents and young adults. 3 studies focused on the association between feeding and eating disorders and NSSI or suicidality. One study examined the risk of suicide in patients with feeding and eating disorders. One study examined self-criticism as a transdiagnostic factor between disordered eating and NSSI. One study looked at socio-demographic and psychological risk factors for suicidal behaviours in patients with feeding and eating disorders. Further details of the studies can be seen in Table 2. The number of studies in the included systematic reviews ranged from 9 to 79, with a total of 275 separate studies included across the 14 systematic reviews. When calculating the total sum of participants, studies repeated across the systematic reviews were excluded. On occasions there were differences in the total number of participants recorded, the lowest reported value and the highest reported value were taken to calculate two values, with the true sum of the participants included lying somewhere within this range (*n* = 2,586,712–2,659,018). Notably, one study conducted by Yao 2016, examined a Swedish birth cohort including individuals born between the 1 st of January 1979, and the 31 st of December 2001 (*n* = 2,268,786). This markedly skews the total number, and when omitting this study, the sum of participants falls significantly (*n* = 390,232–317,935). One systematic review and literature review had been included in studies looked at, however these had been omitted when calculating the total number of participants.

**Table 2:** Characteristics of the included studies

Author (year)	Relationship between EDs and self-harm/suicidality studied	Type of eating disorder	Type of self-harm/suicidality	Review Type	Number of included studies (and study design if included)	Frequency of reported outcomes in meta-analysis (values in parentheses are 95% confidence intervals)	Overall findings	JBI quality score (%)
Amiri, 2023 [30]	Prevalence of non-suicidal self-injury, suicidal ideation, suicide attempts, and death by suicide in a variety of eating disorders	Eating disorders (EDs), including anorexia nervosa (AN), bulimia nervosa (BN), binge eating disorder (BED), avoidant restrictive food intake disorder (ARFID) and eating disorders otherwise not specified (EDNOS)	Non suicidal self-injury, suicidal ideation, suicide attempts, death by suicide	Systematic review and meta-analyses	52 studies	In any eating disorder: Prevalence of NSSI = 40% (33–46%) Prevalence of suicidal ideation = 51% (41–62%) Prevalence of suicide attempts = 22% (18–25%) In bulimia nervosa: Prevalence of NSSI = 44% (34–53%) Prevalence of suicidal ideation = 60% (35–85%) Prevalence of suicide attempts = 25% (19–31%) In anorexia nervosa: Prevalence of NSSI = 29% (21–38%) Prevalence of suicidal ideation = 50% (16–83%) Prevalence of suicide attempts = 17% (13–21%)	A higher prevalence of suicidal ideation is found in eating disorders, followed by non-suicidal self-injury and suicide attempts. Prevalence for NSSI. Suicidal ideation and suicide attempts were higher in patients with bulimia nervosa than anorexia nervosa	81
Conti, 2017 [31]	Relationship between binge-eating disorder and suicidality	Binge-eating disorder	Suicidal ideation, suicide attempts and death by suicide	Systematic Review	17 studies – 12 cross sectional studies and 5 longitudinal studies	N/a	There is a significant association between BED and suicide risk. There is limited research on the effect size of BED on suicide risk. It is unclear whether this is due to comorbid psychopathology, or the commonalities present in BED and other psychiatric conditions	54
Cucchi, 2016 [32]	Lifetime prevalence of non-suicidal self-injury in eating disorder patients	Eating disorders, including anorexia nervosa (AN) and bulimia nervosa (BN)	Non-suicidal self-injury	Systematic review and meta-analysis	29 studies	In any eating disorder: Prevalence of lifetime NSSI = 27.3% (23.8—31.0) In bulimia nervosa: Prevalence of lifetime NSSI = 32.7% (26.9—39.1%) In anorexia nervosa: Prevalence of lifetime NSSI = 21.8% (18.5—25.6%)	There is a high prevalence of NSSI in adolescents and young adults. This correlates positively with history of attempted suicide and negatively with a history of substance abuse	72
Eisenman, 2025 [33]	Co-occurring prevalence of non-suicidal self-injury and disordered eating among adolescents and young adults	Clinical eating disorders and disordered eating	Non-suicidal self-injury	Systematic review	15 studies, cross sectional or longitudinal	N/a	Comorbid disordered eating and NSSI is more prevalent among girls, and those who engage restrictive eating behaviours. Comorbid disordered eating and NSSI is less prevalent in youth than diagnosed eating disorders and NSSI prevalence	91
Goldstein & Govind, 2019 [34]	Socio-demographic and psychological risk factors for suicidal behaviour in individuals with anorexia and bulimia nervosa	Anorexia: restrictive and binging purging subtypes	Suicidal ideation, suicide attempts and death by suicide	Systematic review	38 studies, 36 cross sectional and 2 longitudinal	N/a	Purging behaviours, comorbid disorders (e.g. depression), poor emotional regulation, history of childhood, physical and emotional abuse, pain tolerance, and certain personality and interpersonal features (e.g. perceived burdensomeness) identified as risk factors for suicidality	54
Kirkpatrick, 2024 [35]	Prevalence of non-suicidal self-injury in eating disorder patients	Eating disorders (EDs), including anorexia nervosa: restrictive and binging purging subtypes (AN-R, AN-BP), bulimia nervosa (BN), binge eating disorder (BED), and eating disorders otherwise not specified (EDNOS)	Non-suicidal self-injury	Systematic review and meta-analysis	79 studies	In all eating disorders: Prevalence of NSSI: 34.59% (30.49–38.81%) In anorexia nervosa-—restrictive subtype: Prevalence of NSSI: 24.19% (16.96–30.03%) In anorexia nervosa – binging purging subtype: Prevalence of NSSI: 41.98% (32.25–51.91%) In bulimia nervosa: Prevalence of NSSI:36.97% (30.69–43.46%) In binge eating disorder: Prevalence of NSSI: 21.21% (14.93–28.12%)	There is a higher prevalence of NSSI in people with eating disorders, compared to the general population. The prevalence of NSSI varies depending on the type of eating disorder; OSFED has a relatively high prevalence	91
Mandelli, 2018 [36]	Prevalence of suicide attempts in patients with eating disorders	Eating disorders (EDs), including anorexia nervosa: restrictive and binging purging subtypes (AN-R, AN-BP), bulimia nervosa (BN), binge eating disorder (BED)	Suicide attempts	Systematic review and meta-analysis	43 studies	Risk difference of suicide attempts comparing patients with the binging purging subtype of anorexia nervosa to the restrictive subtype of anorexia: 0.51 Risk difference of suicide attempts comparing patients with restrictive subtype of anorexia nervosa to bulimia nervosa: −0.50 Risk difference of suicide attempts comparing patients with binging purging subtype of anorexia nervosa to bulimia nervosa: −0.63 Risk difference of suicide attempts comparing patients with bulimia nervosa to binge eating disorder: 0.13	A higher proportion of suicide attempts was found in bulimia nervosa than anorexia nervosa The risk of suicide attempts for patients with binge eating disorder appears similar to the risk for patients with AN-R There is a lower proportion of suicide attempts in BED as compared to AN-BP and BN. It may be possible that the binging-purging behaviour is a risk factor for suicidal behaviour in patients with eating disorders	81
Meier, 2024 [37]	Lifetime prevalence of non-suicidal self-injury in children and adolescents with eating disorders	Eating disorders (EDs), including anorexia nervosa: restrictive and binging purging subtypes (AN-R, AN-BP), bulimia nervosa (BN), binge eating disorder (BED)	Non suicidal self-injury	Systematic review and meta-analysis	15 studies	In eating disorders: Prevalence of NSSI: 34.2% (27.5–41.7%) In community samples: Prevalence of NSSI: 26.6% (15.8%—41.2%) In outpatient samples: Prevalence of NSSI: 31.3% (21.7–42.7%) In inpatient samples Prevalence of NSSI: 41.1% (29.5–53.7%) In anorexia nervosa patients: Prevalence of NSSI: 23.3% (16.6–31.8%) In bulimia nervosa: Prevalence of NSSI: 53.2% (42.5–63.6%) In anorexia nervosa – restrictive subtype analysis: Prevalence of NSSI: 15.8% (6.8–32.4%) In anorexia nervosa – binging purging subtype analysis: Prevalence of NSSI: 51.9% (34.4–69%) In bulimia nervosa – subtype analysis: Prevalence of NSSI: 53.7% (27.5–78%)	Prevalence of lifetime NSSI is higher for patients with bulimia nervosa and anorexia nervosa binge eating/purging subtype than anorexia nervosa restrictive type. Prevalence of NSSI is higher in adolescents with an eating disorder than in adults with an eating disorder The meta-analysis is limited by a small number of studies and large heterogeneity between them	72
Pompili, 2004 [38]	Prevalence of suicide in patients with anorexia nervosa	Anorexia nervosa (AN)	Suicide	Systematic review and meta-analysis	9 studies	Mean number of expected suicides in a population of 100,000 with anorexia nervosa: 24 Standard deviation for a population of 100,000 suffering from anorexia nervosa: 19 Mean number of suicides expected in a general population of 100,000: 3	Higher rates of suicide occur in populations with anorexia nervosa than the general population Anorexia nervosa alone may be risk factor for suicide	0
Preti, 2010 [39]	Risk of suicide in patients with eating disorders	Eating disorders (EDs), including anorexia nervosa (AN), bulimia nervosa (BN), binge eating disorder (BED)	Suicide	Systematic review and meta-analysis	59 studies	Risk rate for suicide in patients with anorexia nervosa in a fixed model: 34.99 (29.66–41.27) Risk rate for suicide in patients with anorexia nervosa in a random model: 35.37 (26.39–47.40) Risk rate for suicide in patients with bulimia nervosa in a fixed and random model: 17.4 (9.3–32.3)	The risk of suicide in patients with anorexia nervosa is higher than the general population. This prevalence of suicide in patients with anorexia nervosa has also decreased in recent years The risk of suicide is also high for patients with bulimia nervosa, although the risk of not as high as for those with anorexia nervosa	45
Sesboüé, 2025 [40]	Association between non- suicidal self- injury and anorexia	Anorexia nervosa: restrictive and binging purging subtypes (AN-R, AN-BP)	Non-suicidal self-injury	Systematic Review	33 studies	N/a	NSSI and anorexia nervosa are frequently associated with each other, making management of these patients more complex. Only a diagnosis of anorexia nervosa with binge eating/purging behaviours predicts NSSI occurring during the eating disorder	63
Smith, 2014 [41]	Association between suicidal thoughts, and behaviours and eating disorders	Eating disorder diagnosis, some specific	Non-suicidal, self-injury, suicidal ideation, suicide attempts, death by suicide	Systmatic review and meta-analysis	14 studies	wOR of suicide attempts in patients with eating disorders: 1.91 (1.48–2.48) wOR of suicide attempts in patients with eating disorder diagnosis: 2.19 (1.55–3.09) wOR of suicide attempts in patients with eating disorder symptoms: 1.81(1.35–2.42) wOR of suicide attempts in patients with eating disorders:	Eating disorders are weak risk factors for suicide attempts	55
Sohn, 2023 [42]	Prevalence of non-suicidal self-injury, suicidal thoughts, and behaviours in individuals with eating disorders, relative to healthy and psychiatric controls	Confirmed eating disorder diagnoses or self-reported symptoms, including anorexia nervosa (AN), bulimia nervosa (BN), binge eating disorder (BED)	Non-suicidal self-injury, suicidal ideation, suicide attempts and death by suicide	Systematic review and meta-analysis	32 studies	Comparing patients with eating disorders to healthy controls: OR of NSSI: 6.85 (3.60–13.04) OR of suicidal ideation: 3.63 (2.43–5.41) OR of suicide attempts: 5.16 (4.27–6.24) Comparing patients with eating disorders to psychiatric controls: OR of NSSI: 2.74 (1.49–5.06) OR of suicidal ideation: 3.10 (2.01–4.78) OR of suicide attempts = 1.37 (0.37–4.99) Comparing types eating disorder to controls: OR of suicide attempts in anorexia nervosa compared to combined healthy and psychiatric control groups = 3.19 (2.04–4.99) OR of suicide attempts in anorexia nervosa compared to healthy controls = 5.55 (4.02–7.65) OR of suicide attempts in anorexia nervosa compared to psychiatric controls = 0.90 (0.38–2.11) OR of suicide attempts in bulimia nervosa compared to combined controls = 5.93 (3.81–9.23) OR of suicide attempts in binge eating disorder nervosa compared to combined controls = 3.37 (2.68–4.23) OR of completed suicide in bulimia nervosa compared to combined controls = 2.93 (0.78–10.93)	There is increased prevalence of NSSI, suicidal ideation and suicide attempts in patient groups with eating disorders than those without Individuals with eating disorders are 3 times more likely than a healthy or psychiatric control group to experience suicidal ideation The rate of suicide attempts or death by suicide were not higher in groups with eating disorders than groups without eating disorders	91
Zelkowitz, 2018 [43]	Examines self-criticism as a transdiagnostic process in non-suicidal self-injury and disordered eating	Disordered eating	Non-suicidal self-injury	Systematic review and meta-analysis	15 studies related to self-criticism and non-suicidal self-injury, 24 studies related to self-criticism and disordered eating	Self-criticism and NSSI: Correlation coefficient for the relation of self-criticism and NSSI in clinical samples: 0.47 (0.33–0.59) Correlation coefficient for the relation of self-criticism and NSSI in community samples: 0.36 (0.25–0.45) Self-criticism and disorder eating: Correlation coefficient for the relation of self-criticism and disordered eating: 0.40 (0.34–0.45) Correlation coefficient for the relation of self-criticism and purging: 0.51 (0.42–0.58) Correlation coefficient for the relation of self-criticism and binging: 0.38 (0.33–0.43) Correlation coefficient for the relation of self-criticism and restriction: 0.32 (0.24–0.39)	Both disordered eating and NSSI had a large and significant correlation to self-criticism. This varied between forms of disordered eating	72

Several studies synthesised the prevalence of feeding and eating disorders and NSSI [30, 32, 35, 42] and/or suicidal behaviour in adults [30, 31, 36, 38, 42]. A higher prevalence of suicide attempts was also found in patients with anorexia nervosa when compared to the general population [38].

Upon comparing different types of eating disorder, a higher proportion of suicide attempts were found in patients with bulimia nervosa than anorexia nervosa. Moreover, the proportion of suicide attempts is lower in patients with BED, compared to patients with the AN-BP or BN [36]. When looking at the prevalence of NSSI, suicidal ideation, suicide attempts and completed suicide amongst patients with feeding and eating disorder one review found suicidal ideation was the most common. This was followed by NSSI, and then suicide attempts, with prevalence rates of 51% (CI 41–62%), 40% (CI 33–46%) and 22% (CI 18–25%), respectively [30]. The prevalence of suicide in patients with AN has also been found to have decreased in recent years [39]. Additionally, Sohn, 2023 found increased risk of NSSI, suicidal ideation and suicide attempts, but not completed suicide, in individuals with feeding and eating disorders compared to healthy control groups. Individuals with feeding and eating disorders are three times more likely to experience suicidal ideation than healthy or psychiatric control groups [42]. A higher risk of suicide was also identified in patients with AN and BN, with the risk in patients with AN being higher than for those with BN [39]. Similarly, the risk of suicide in adolescent populations was also higher for those with feeding and eating disorders [34]. A review by Conti focuses on the relationship between suicidality and binge eating disorders, and found suicidality (referring to suicidal ideation, suicide attempts and death by suicide) was higher in adolescent groups with feeding and eating disorders than their adult counterparts [31]. One review studied whether eating disorders influenced the risk of suicide attempts, and concluded eating disorders were weak risk factors for suicide attempts.

#### Risk of bias assessment

Both authors independently applied the JBI critical analysis tool to the 14 reviews in this study. Results were then compared with a 94.8% agreement between authors. Table 3 shows the JBI analysis conducted by reviewer AB (see appendix for the JBI analysis conducted by reviewer RW). JBI analysis scores for the included reviews ranged from 0/11 (0%) to 10/11 (91%), and with an average of 69.9%. 11 out of 14 studies did not assess the likelihood of publication bias, however this is usually associated with randomised control trials, particularly those focusing on effectiveness of treatment. These reviews typically influence clinical decision making or policies, therefore it is important to be aware of any potential overestimation of a treatment’s effects. As the included systematic reviews mainly focused on the association between feeding and eating disorders, self-harm and/or death by suicide assessing the likelihood of publication bias was not necessarily relevant to the review. The most overlooked aspects in the included studies were: (1) appropriate criteria for appraising the studies, (2) a critical appraisal of studies conducted by two independent reviewers.

**Table 3:** A JBI analysis of the included systematic reviews (conducted by reviewer AB)

	Amiri, 2023	Conti, 2017	Cucchi, 2016	Eisenman, 2025	Goldstein, 2019	Kirkpatrick, 2024	Mandelli, 2018	Meier, 2024	Pompili, 2004	Preti, 2010	Sesboüé, 2025	Smith, 2018	Sohn, 2023	Zelkowitz, 2018
Is the review question clearly and explicitly stated?	Yes	Yes	Yes	Yes	Yes	Yes	Yes	Yes	No	Yes	Yes	Yes	Yes	Yes
Were the inclusion criteria appropriate for the review question?	Yes	Yes	Yes	Yes	Yes	Yes	Yes	Yes	Unclear	Yes	Yes	Yes	Yes	Unclear
Was the search strategy appropriate?	Yes	Yes	Yes	Yes	Yes	Yes	Yes	Yes	No	Yes	Yes	Yes	Yes	Yes
Were the sources and resources used to search for studies adequate?	Yes	Yes	Yes	Yes	Yes	Yes	Yes	Yes	No	No	Yes	Yes	Yes	Yes
Were the criteria for appraising studies appropriate?	Yes	No	No	Yes	No	Yes	Yes	No	No	No	No	No	Yes	No
Was critical appraisal conducted by two or more reviewers independently?	No	Yes	No	Yes	No	Yes	Yes	No	No	No	No	No	Yes	No
Were there methods to minimize errors in data extraction?	Yes	Unclear	Yes	Yes	No	Yes	No	Yes	No	No	No	No	Yes	Yes
Were the methods used to combine studies appropriate?	Yes	Yes	Yes	Yes	Yes	Yes	Yes	Yes	No	Yes	Yes	Yes	Yes	Yes
Was the likelihood of publication bias assessed?	No	Unclear	Unclear	No	No	No	Yes	No	No	Yes	No	No	No	Yes
Were recommendations for policy and/or practice supported by the reported data?	Yes	Yes	Yes	Yes	Yes	Yes	Yes	Yes	N/a	N/a	Yes	N/a	Yes	Yes
Were the specific directives for new research appropriate?	Yes	No	Yes	Yes	Yes	Yes	Yes	Yes	No	Yes	Yes	Yes	Yes	Yes
Score out of 11	9	7	8	10	7	10	10	8	0	5	7	7	10	8
Percentage	81	63	72	91	63	91	91	72	0	45	63	63	91	72

**Fig. 3:**
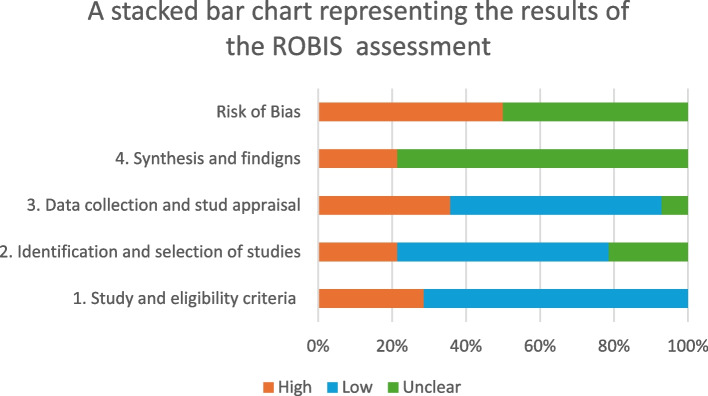
A stacked bar chart representing the results of the ROBIS assessment

A ROBIS assessment was also conducted by author AB, the results of which are shown in Table 4. Overall 7 of the included studies showed high risk of bias, 2 of which demonstrated a high risk of bias throughout all 4 domains. It is important to acknowledge the risk of bias when interpreting results from studies with high levels of concern. Areas which were often overlooked included (1) not registering with a protocol, (2) no attempt to search for unpublished papers, (3) no efforts made to minimise bias in data extraction (Fig. 3).

**Table 4:** A ROBIS analysis of the included systematic reviews (conducted by reviewer AB)

Study	Domain 1: study eligibility criteria	Domain 2: Identification and selection of studies	Domain 3: data collection and study appraisal	Domain 4: synthesis and findings	Risk of bias
Conti et al	Low	Low	Low	Low	Low
Kirkpatrick et al	Low	Low	Low	Low	Low
Sesboüé et al	Low	Low	Unclear	High	High
Cucchi et al	Low	Low	Low	Low	Low
Sohn et al	Low	Low	Low	Low	Low
Amiri et al	High	Unclear	High	Low	High
Goldstein et al	High	High	High	High	High
Meier et al	Low	Low	Low	Low	Low
Eisenman et al	Low	Low	Low	Low	Low
Zelkowitz et al	Low	High	High	Low	High
Mandelli et al	Low	Low	Low	Low	Low
Smith et al	High	Unclear	Low	Low	High
Preti et al	Low	Unclear	High	Low	High
Pompili et al	High	High	High	High	High

### Feeding and eating disorders and NSSI in adults

There is evidence that non-suicidal self-injury is more common in populations with feeding and eating disorders than the general population [35]. This is supported by a systematic review and meta-analysis conducted by Sohn et al., which reported a pooled odds ratio of 6.85 (3.60–13.04) for NSSI in feeding and eating disorders when compared to healthy controls, and 2.74 (1.49–5.06) when compared to a psychiatric control group. Furthermore, the prevalence of NSSI within feeding and eating disorder patients are higher in women, similarly to the prevalence of NSSI in the general population [35] and a systematic review conducted by Cucchi et al. found participants from speciality feeding and eating disorder or residential treatment settings were 2.6 more likely to report NSSI.

Four studies looked at the prevalence of NSSI in patients with feeding and eating disorders [30, 32, 35, 42]. Three studies found a higher prevalence rate in patients with bulimia nervosa than those with anorexia nervosa. A systematic review by Kirkpatrick was the only study to include the restrictive and purging and binging subtype of anorexia nervosa, and instead found the prevalence of NSSI was highest in AN-BP (41.98 [CI 32.25–51.95%]). Bulimia nervosa was second highest (36.97% [30.69–43.46%]), followed by AN-R (24.19% [16.96–30.03%]) and then BED (21.21% [14.93–28.12%]). Although no meta-analysis included OSFED, there is evidence for a high prevalence of NSSI [35].

Individuals with feeding and eating disorders who engage in NSSI, especially binging or purging, are. likely engage in a larger range of NDDI methods, associated with a persistent course of NSSI and suicidal behaviour [42]. Age, body mass index, treatment time, patient diagnoses and age of feeding and eating disorder were all not significant in onset of NSSI in patient with feeding and eating disorders. The duration of the feeding and eating disorder, however, was found to contribute to the risk of NSSI onset in individuals with feeding and eating disorders [43].

There is evidence that NSSI often occurred during moments of intense internal tension, commonly after anxiety inducing situations, such as mealtimes, feelings of satiety or as a means of self-punishment [43]. Moreover, NSSI could be used as a coping strategy for suicidality, as suicidal ideation and attempts are less frequent in patients who engage in more frequent NSSI [43].

A study by Sesboue found no significant difference was found between the type of NSSI or body area and subtype of anorexia nervosa. Additionally, adverse childhood experiences are commonly found in patients with anorexia nervosa who engage in NSSI, as compared to acute stressful events occurring immediately before the onset of NSSI. This suggests a more ‘chronic’ emotional dysregulation. The same review also found these patients were more likely to experience dissociative episodes, impulsivity, anxiety, depression, and poorer self-esteem and require more complex management. When comparing the restrictive and binging-purging subtype, patients with AN-R who engaged in NSSI had a higher prevalence of psychiatric comorbidities, including major depressive episodes, anxiety disorders and personality disorders. On the other hand, patients with AN-BP were more likely to have a childhood history of sexual abuse, suicide attempts, suicidal ideation, and higher impulsivity.

The effects of NSSI on the course of anorexia nervosa were also investigated in this study. Patients with AN-BP were more likely to experience suicidal ideation and attempt suicide, after engaging in NSSI, whereas patients with AN-R were more likely to experience depression, anxiety, suicidal ideation, lower weekly weight gain and longer treatment duration. Only AN-BP was found to predict NSSI throughout the duration of the feeding and.

### Feeding and eating disorders and NSSI in adolescents

A higher prevalence of NSSI was found in adolescents than adults, in one review [40]. This varies between subtypes, with a higher occurrence in patients with bulimia nervosa, then the binging purging subtype of anorexia nervosa, and then the restrictive subtype [36, 40]. Although the study by Meier did not find this in adult populations, there is evidence from the study by Kirkpatrick that prevalence rate differs based on the type of feeding and eating disorder, with AN-BP having the highest prevalence, followed by BN and then AN-R.

The occurrence of NSSI was lower in populations with disordered eating compared to those with feeding and eating disorders [36], although the relationship between disordered eating or feeding and eating disorders and NSSI was found to be bidirectional.

Two studies looked at the risk factors for NSSI in adolescent populations with eating disorders [35, 36]. A higher prevalence of NSSI also correlated with a history of substance abuse and attempted suicide, in adolescent populations with feeding and eating disorders, indicating more complex psychopathologies [35]. Childhood maltreatment is a significant risk factor for NSSI, and recent sexual abuse mean NSSI was seven times more likely. The study also suggested the influence of social dysfunction is stronger in adolescents as they are more sensitive to social evaluation from peers, increasing risk of engaging in NSSI.

### Feeding and eating disorders and suicidal ideation in adults

A review by Sohn et al. compared suicidal ideation in a group with feeding and eating disorders to a control group with no psychiatric conditions and a group with psychiatric conditions. A random-effects meta-analysis was carried out and found an increased prevalence of suicidal ideation in individuals with feeding and eating disorders when compared with a control group with no psychotic conditions (OR = 3.63 [95% CI: 2.43–5.41]). This included one study which assessed adolescents (aged 12–18y years old). When repeating the analysis, excluding the youth sample, similar results were found (OR = 2.99 [95% CI: 2.11–4.22]). When comparing the prevalence of suicidal ideation with the group with psychiatric conditions, patients with eating disorders were again 3 times more likely to experience suicidal ideation, (OR = 3.10 [95% CI: 2.01–4.78]). This is partially supported by one study in the systematic review conducted by Goldstein & Govind, which found patients with bulimia nervosa are more likely to experience suicidal ideation than groups with no psychopathologies. However, no significant increase in likelihood of suicidal ideation was found in groups with binge eating disorder or other psychopathologies was also found.

Additionally, a higher prevalence of suicidal ideation was found in patients with binge eating disorder who also had alexithymia [35].

### Feeding and eating disorders and suicidal ideation in adolescents

When looking at patients with binge-eating disorders, the prevalence of suicidal ideation was higher in adolescents with the condition, compared to patients without and more common amongst girls [35]. There is also evidence that as the severity of binge eating disorder increases, the prevalence of suicidal ideation also increases. Unlike in adult populations, the occurrence of suicidal ideation differs between types of feeding and eating disorders; patients with bulimia nervosa demonstrate a higher frequency of suicidal ideation compared to those with anorexia nervosa [37].

In adolescents with binge eating disorder, it was found that the onset of the feeding and eating disorder precedes suicidal ideation, planning and attempting whereas the reverse is true for adults with binge eating disorder. In adults, binge-eating disorder may manifest as a way for coping with suicidality [35].

### Feeding and eating disorders and suicide attempts in adults

There is evidence that the prevalence of suicide attempts was most common in patients with bulimia nervosa, then anorexia binging-purging subtype, anorexia restrictive subtype, then binge-eating disorder [37]. Additionally, a meta-analysis done by Amiri found the prevalence of suicide attempts in feeding and eating disorder patients was 22% (CI 18–25%). Upon subtype analysis looking at anorexia nervosa and bulimia nervosa only, a higher prevalence of 25% (CIO 19–31%) was found in patients with bulimia nervosa, compared to 17% (CI 13–21%) in anorexia nervosa.

A higher proportion of suicide attempts were seen in patients with bulimia nervosa than anorexia nervosa. Additionally, a risk difference of 0.51 was calculated for suicide attempts in patients with the binging-purging subtype of anorexia nervosa compared to patients with the restrictive subtype of anorexia nervosa. This is similar to the risk difference of 0.50 calculated when comparing patients with bulimia nervosa to the restrictive subtype of anorexia nervosa. Patients with binge eating disorder appeared to have a similar level of risk to patients with the restrictive subtype of anorexia nervosa [36]. A study by Smith examined the risk of an eating disorder diagnosis on the risk of suicide attempt and found a significant increase in risk of attempt but not completed suicide [41].

There is evidence that patients with feeding and eating disorders who had attempted suicide had a maladaptive attitude towards death [37], which was not present in patients with eating disorder who had not attempted suicide previously.

For patients with anorexia nervosa, there is evidence that depression contributes more to the risk of making a suicide attempt, than the condition, anorexia nervosa itself [37]. The same systematic review also found that people with bulimia nervosa and MDD were more likely to have experienced substance abuse, parental violence, unemployment and physical or sexual violence. Emotional and sexual abuse during childhood, specifically, was found to increase risk of these patients attempting suicide [37]. Similarly to suicidal ideation, a higher prevalence of suicide attempts was found in patients with binge eating disorder who also had alexithymia, and with some evidence that standard dialectal therapy helped to reduce the risk of patients attempting suicide [35].

### Feeding and eating disorders and completed suicide in adults

A review by Pompilli concluded that the rates of suicide in patients with anorexia nervosa are higher than the general population [38]. Similar findings were reported in the review by Preti, however it also found that the rate of suicide found in these patients has decreased in recent years. Additionally, the rate of suicide decreased as the length of the follow up period increased, however the converse was true for bulimia nervosa, where the rate of suicide was positively related to the length of the follow up period [39].

The risk of suicide for patients with bulimia nervosa was found to be higher than the general population, but lower than anorexia nervosa [39].

### Risk factors for self-harm and suicidality with feeding and eating disorders

A systematic review by Goldstein & Govind focused on sociodemographic and psychological risk factors for suicidal behaviour in patients with anorexia or bulimia nervosa. There is limited research on the risk of background factors for suicidal behaviours in patients with feeding and eating disorders. There is some evidence to suggest experiences of physical and sexual abuse during childhood correlate to patients with bulimia nervosa attempting suicide in adulthood, however bulimia nervosa itself has not been found to be associated with suicide attempts. Additionally, there is evidence for a common genetic influence on suicidality and that patients with a full sibling or cousin with any feeding having an increased risk of attempting suicide themselves [34].

There is evidence that higher levels of overall psychological distress increase the risk of suicidal ideation and self-harm, with adolescents, especially girls, being particularly vulnerable. Feelings of sadness or anxiety were commonly associated with adolescents who experiences suicidal ideation and worthlessness with attempting suicide. Bulimia nervosa was also found to be more frequent amongst older adolescents and adults, and the review suggests it may act as a mediating factor in the associations between anorexia nervosa, bulimia nervosa and suicidal attempts [34].

All feeding and eating disorders were found to be characterised by negative self-image. This was also associated with prior suicide attempts in patients with the restrictive subtype of anorexia nervosa and EDNOS and later suicide attempts or completed suicide in women with bulimia nervosa.

The review also reports that deaths by suicide among individuals diagnosed with feeding and eating disorders occurred due to the highly lethal suicide attempt made, rather than compromised physical health due to their feeding and eating disorder. This suggests that an acquired capability for suicide is an important risk factor. Joiner’s Interpersonal theory of suicidal behaviour states that individuals may become habituated to pain over the course of their illness, increasing their capability for suicide and lead to death from highly lethal suicide attempts. A study conducted by Selby in 2010 [44] found that painful and harmful behaviours such as laxative abuse and NSSI mediated the relationship between AN-BP and extreme suicidal behaviour. Furthermore, overexercise was positively related to pain insensitivity and was found to be a predictor of suicidal behaviour in women with bulimia nervosa, suggesting behaviours from the feeding and eating disorder can involve painful and provocative experiences which contribute towards an acquired capability for suicide.

Some research has examined the association of perceived burdensomeness and thwarted belongingness to feeding and eating disorders and suicidality, both as factors described in Joiner’s IPTS. More severe symptoms of a feeding and eating disorder were associated with higher levels of perceived burdensomeness and thwarted belonginess, which were both associated with a higher risk of suicide. Current body dissatisfaction and fasting specifically were related to increased suicidal ideation through a higher sense of perceived burdensomeness, and this factor was found to more frequently explain the association between feeding and eating disorders and suicide than thwarted belongingness.

The review found depression and aggressiveness were both important antecedents and correlates of suicidal ideation and contemplation in both adolescents and adults with feeding and eating disorders. A cross sectional study among adolescents, that explored the relationship between anorexia nervosa, depression, and suicidal thoughts, found depression may have a larger contribution towards suicidal thoughts than anorexia nervosa, suggesting a more robust association between depression and suicidal thoughts. Major depression and the migration from AN-R to AN-BP were also risk factors for suicide in patients with feeding and eating disorders and depressive symptoms tend to be more severe in patients who had attempted suicide. Both onset of major depression and the switch from restrictive to binging/purging subtype of anorexia nervosa was more frequent before suicide attempts.

Poor emotional regulation was associated with an increased risk of engaging in purging behaviours and, along with increased impulsivity, suicide. Patients with eating disorders and suicidal behaviour were also more likely to engage in self harm. Body dissatisfaction and disturbances, less repulsion from death and a greater attraction to death were associated with an increased risk of suicide attempts in patients with feeding and eating disorders. Body dissatisfaction and disturbances however was not found to predict suicidal ideation or attempting suicide, suggesting it has long-term links to suicidal behaviour.

### Self-criticism as a transdiagnostic link

Self-criticism as a transdiagnostic link between disordered eating behaviours and NSSI.

One review focused on self-criticism as a transdiagnostic link between disordered eating and NSSI [43]. Both disordered eating and NSSI had large correlations with self-criticism, and this varies depending on the type of disordered eating behaviour; it was most highly correlated with purging, then mixed (binging, purging and restriction) and the smallest (although still significant) binging and restriction. An odds ratio of 0.42 (CI 0.36–0.48) was seen in adult samples and 0.33 in adolescent samples (0.22–0.39).

## Discussion

This umbrella review synthesised 14 systematic reviews (with meta-analyses conducted for 10 of them), regarding the association, prevalence, and risk factors of feeding and eating disorders with self-harm and suicidality. This umbrella review aimed to provide a top-level overview on the relationship between feeding and eating disorders and suicidality. 5 studies conducted a meta-analysis looking at the prevalence of self-harm and suicidality in patients with feeding and eating disorders. There is evidence of a statistically significant elevated prevalence and risk of self-harm, suicidal ideation, and suicidal attempts, when compared to healthy counterparts. An increased rate of completed suicide was also seen in patients with feeding and eating disorders, however, there was mixed evidence for whether having a feeding and eating disorder increased risk of completed suicide, with one study in support [41] and two studies against [39, 42]. There is consistent evidence that prevalence of these psychopathologies was higher in adolescents compared to adults, especially in girls. The effect size of potential risk factors for self-harm and suicide was only studied in one meta-analysis, which looked at self-criticism as a transdiagnostic factor. Therefore, there is only quantitative evidence for the strength of the association between self-criticism and NSSI and self-criticism and disordered eating, both of which were found to be statistically significant. The magnitude of other risk factors in causing NSSI or suicidality remains unclear and is an area for further research. This discussion explores how this study’s findings relate to the IMV model and summarises the interplay between feeding and eating disorders and NSSI and suicidality.

The IMV theory provides a framework for how an individual can progress to experiencing suicidal ideation and engaging in suicidal behaviours, from initial feelings of defeat and hopelessness. With the background risk for suicidal ideation or attempts initially influenced by pre-motivational factors, the IMV theory states threat to self, motivational and volitional moderators then strengthen the progression through the stages of feeling defeated, entrapment, suicidal ideation and attempting suicide respectively. Further research is needed on associated risk factors for suicidality in feeding and eating disorder patients and this framework provides a suggestion for the factors to be studied in this research. Additionally, comparing the strength of these moderators for the progression to suicidal ideation in patients with feeding and eating disorders and patients without, may provide a better understanding as to why there is increased prevalence of suicidality in patients with feeding and eating disorders and the reasons behind this. This umbrella review has identified that the risk of a suicide attempts is similar for patients with AN-BP or BN and is higher than the risk of suicide in patients with AN-R or BED [38], suggesting the act of engaging in purging is a risk factor for suicide attempts. AN-BP is the only type of feeding and eating disorder found to be independently associated with suicidal ideation and attempts, and higher levels of impulsivity are also associated with AN-BP [40]. Impulsivity is a volitional moderator in the IMV model and is posited to influence the progression of suicidal ideation to suicide attempts. Recognising impulsivity as a significant factor for suicide attempts for patients with AN-BP can inform clinical practice. Educating at risk patients about the role impulsivity plays in suicide attempts and putting in place safety plans that acknowledge and provide measures to cope with increased feelings of impulsivity can help to safeguard patients more effectively. Additionally, psychotherapy targeting increased feeling of impulsivity can further help individuals to manage feelings of impulsivity in the longer term, helping to decrease likelihood of impulsive of future suicide attempts.

Although the available longitudinal evidence available on self-harm and suicidality in patients with feeding and eating disorders is limited, a key finding from this review was that the onset of binge eating disorder typically preceded the onset of suicidal ideation or attempting suicide in adolescents. On the other hand, the reverse was typically true for adults [34]. Feeding and eating disorders are known to have unstable diagnoses, with clinical features migrating between subtypes over time and often found to be comorbid with other diagnosis, such as anxiety or depression [45], suggesting a transient nature. Additionally, feeding and eating disorder behaviours can serve multiple psychological functions including emotional regulation [46], self-punishment [47], control [48], or aligning to societal body ideals [49]. The evidence that onset of binge eating disorder typically precedes onset of self-harm and/or suicidality in adolescents and the reverse for adults, indicates that the psychological purpose of the behaviours may differ due to age. Similarly, a range of factors, including those psychological, social and environmental nature, contribute to the onset of self-harm in individuals, and specific factors can vary between individuals [50]. It is possible that increased sensitivity to external pressure regarding body size and shape evaluation [51], and the development of positive or negative body image [52] that occurs during adolescence, results in feeding and eating disorder behaviours serving more of a social reinforcement role for adolescent patients. For adults who develop feeding and eating disorders after experiencing self-harm or suicidality, it is possible their previous history of self-harm and suicidality influences the function of their eating disorder behaviour. Punishment is a key theme of self-harm [53], and feeding and eating disorder behaviours could act to redirect the method of self-punishment, with it potentially being easier to hide or more socially acceptable than self-harm. Two key points emerge from this: feeding and feeding and behaviour may be used to serve different psychological functions, specific to the individual, and a better understanding of the timeline for the onset of feeding and eating disorders and self-harm and suicide is needed. The specific reasons a patient engages in feeding and eating disorder and self-harm behaviours needs to be explored with each patient, and then used to personalise treatment, in order to address the root causes and increase efficacy of treatment. Additionally, it is highly probable that either diagnosis influences the onset and reason for engaging in the other behaviour. More longitudinal research to understand which condition is more likely to develop first is critical to then understand the interplay between them.

### Clinical implications

For clinicians, the high prevalence of self-harm and suicidality in feeding and eating disorder patients means awareness and routine screening is needed in patients with feeding and feeding and eating disorders, especially in a primary care setting. Binging and purging behaviours, in particular, had a higher association with suicidal ideation and attempts and is the relationship has been found to be mediated by self-harm [41]. Early and routine screening for suicidal ideation of behaviours allows to a patient to be appropriately risk assessed and opportunity for early intervention. There are a range of screening tools available for assessing suicide risk, with the Beck Scale for Suicide Ideation and the Colombia – Suicide Severity Rating Scale (C-SSRS) being two of the most common measures used [54]. Out of the available scales, the C-SSRS can be used globally, with adaptation to and linguistic validations for over 100 country-specific languages [55]. The scale also separates ideation from behaviours, measuring the intensity of each. However, there is still need for a new screening tool which covers all psychopathological aspects of suicidality [56].

Migration between types of feeding and eating disorders is common [57], and it is important to monitor this within patients. Understanding that feeding and eating disorder behaviours can stem from different causes can allow for individualised, targeted care and allow for the root cause of the behaviours to be addressed and support longer-term recovery.

### Areas of further research

As mentioned previously, more research is needed comparing relevant factors suggested by the IMV model, in the progression of suicidality between patients with feeding and eating disorders and patients without. This allows for the progression of suicidality to be compared between these two groups, providing clarity on any differences in the progression of suicidality in patients with feeding and eating disorders. This can then tailor suicide intervention and prevention strategies for patients with eating disorders, helping to address the higher prevalence found in this population.

Quantitative data regarding the frequency and distribution of events throughout the patients’ life was consistently lacking amongst the systematic reviews included in this study. More longitudinal research is needed looking at the onset of feeding and eating disorders and suicidality, to understand if and how onset of one of these conditions influences the other. It is likely there is not one clear timeline, due to the multifactorial influence for both conditions, however it remains largely unclear how these conditions interact, and data for is needed.

None of the studies found by the searches conducted in this review researched the relationship between self-harm and/or suicidality and PICA, ARFID and rumination regurgitation disorder. This may be explained by the recent inclusion of these types of feeding and eating disorders in the ICD 11 and is an area for future research.

Eating disorders and psychotic illness comorbidity ranges from ranging from transient psychotic symptoms to comorbid schizophrenia and anorexia nervosa [58] and has multiple patterns of psychopathology [58].In the general population, psychotic symptoms are associated with a higher risk of self-harm [59] and suicidality [60]. While there is currently mixed evidence for whether psychotic symptoms occur at higher prevalence in patients with feeding and eating disorders, it is important to consider the implications psychotic symptoms may have on the risk of self-harm and suicidality in patients with feeding and eating disorders. Psychosis is a set of symptoms results in a loss of contact with reality, with patients often experiencing by hallucinations, delusions, and cognitive impairment [56]. For patients with psychotic symptoms or at high risk of psychosis, disordered eating behaviours may interact with these symptoms in numerous ways, including serving as a form of control form a loosening grip on reality or be a direct result of command hallucinations. Patients with feeding and eating disorders commonly have overvalued beliefs of body image, presenting as delusion-like beliefs in severe cases [61]. The association and interaction between feeding and eating disorders and psychotic behaviours, both in terms of self-harm and suicidality and in general, is an area for further research. It is also important for clinicians to be aware of the increased risk of self-harm and suicidality present in feeding and eating disorder patients with psychotic symptoms, as the presence of psychotic symptoms may indicate severity of the presentation and may alter the mechanism behind the risk of self-harm or suicidality.

There was limited research on death by suicide, with studies generally focusing on NSSI, suicidal ideation or suicide attempts. Death by suicide remains a significant cause of mortality in patients with feeding and eating disorders, and further investigation into this allows for factors that may be missed when researching suicide attempts, e.g. lethality of method, and their significance, to be explored.

### Strengths

This umbrella review provides a broad, top-down level analysis, with a significant number of participants (*n* = 2,478,721–2,531,594) included in the umbrella review. A large volume of evidence has been synthesised in this review and findings from systematic reviews have been compared. Findings between systematic reviews largely align, strengthening their reliability. Areas with contrasting evidence, such as whether the risk of suicidal ideation is increased in patients with feeding and eating disorders compared to control psychiatric groups, have been highlighted as areas of research that remain unclear.

### Limitations

Due to the nature of umbrella reviews in providing a top-level synthesis, the inclusion criteria required studies to be systematic reviews. Research conducted by primary studies, that have not then been incorporated into systematic reviews, therefore have not been included in the synthesis of information conducted by this umbrella review. It is possible some of the gaps in research identified by this study have been covered in these reviews.

Within the included studies, populations were conducted in high income, Western countries and had entirely or largely female populations. Although, men have been historically underrepresented in feeding and eating disorder research, men account for approximately 1 in 4 presentations of anorexia and bulimia nervosa [62, 63]. There is a lack of research into mental health in low- and middle-income countries, possibly explained by the scarcity of mental health research resources in these countries, especially in countries across Asia and Africa [64]. The population characteristics of the included studies limit the generalisability of these results to non-Western, lower income countries and male populations. Further research within these populations is needed, in order to assess whether these findings remain for these populations (Table 5 in Appendix).

The JBI method for assessing bias in the included studies is subjective and crude; designed for broad use across different study designs, limiting the quality of its assessment. Additionally, due to time constraints, when conducting the JBI quality assessment, areas of uncertainty were unable to be clarified with the author, limiting the assessment to the included information in the report.

Finally, software, such as Covidence, was not utilised when screening the literature.

## Conclusion

This umbrella review includes that self-harm and suicidality is significantly associated with feeding and eating disorders. Genetic, social, and environmental factors contribute to suicidality in patients with feeding and eating disorder. Self-criticism was also identified as a significant transdiagnostic factor. In line with the IMV theory, behaviours found in feeding and eating disorders contribute to the onset suicidal ideation and suicide attempts. The aetiology of feeding and eating disorder behaviours and self-harm may vary between individuals, and it is important for clinicians to recognise this and tailor psychotherapy treatments accordingly.

## Data Availability

All data generated or analysed during this study are included in these published articles: 1. Amiri S, Khan MA. Prevalence of non-suicidal self-injury, suicidal ideation, suicide attempts, suicide mortality in eating disorders: a systematic review and meta-analysis. Eat Disord. 2023 Sep 3;31(5):487-525. doi: 10.1080/10640266.2023.2196492. Epub 2023 Apr 6. PMID: 37021980. 2. Conti C, Lanzara R, Scipioni M, Iasenza M, Guagnano MT, Fulcheri M. The Relationship between Binge Eating Disorder and Suicidality: A Systematic Review. Front Psychol. 2017 Dec 5;8:2125. doi: 10.3389/fpsyg.2017.02125. 3. Cucchi A, Ryan D, Konstantakopoulos G, Stroumpa S, Kaçar AŞ, Renshaw S, Landau S, Kravariti E. Lifetime prevalence of non-suicidal self-injury in patients with eating disorders: a systematic review and meta-analysis. Psychol Med. 2016 May;46(7):1345-58. doi: 10.1017/S0033291716000027. Epub 2016 Mar 8. PMID: 26954514. 4. Eisenman SR, Jackson I, Hudson LD, Vázquez-Vázquez A. The Co-Occurring Prevalence of Non-Suicidal Self-Injury and Disordered Eating Among Adolescents and Young Adults: A Systematic Review. J Adolesc Health. 2025 Mar https://pubmed.ncbi.nlm.nih.gov/40057885/. 5. Goldstein A, Gvion Y. Socio-demographic and psychological risk factors for suicidal behavior among individuals with anorexia and bulimia nervosa: A systematic review. J Affect Disord. 2019 Feb 15;245:1149-1167. doi: 10.1016/j.jad.2018.12.015. Epub 2018 Dec 10. PMID: 30699859. 6. Kirkpatrick RH;Breton E;Biorac A;Munoz DP;Booij L; (no date) Non-suicidal self-injury among individuals with an eating disorder: A systematic review and prevalence meta-analysis, The International journal of eating disorders. U.S. National Library of Medicine. 7. Mandelli L, Arminio A, Atti AR, De Ronchi D. Suicide attempts in eating disorder subtypes: a meta-analysis of the literature employing DSM-IV, DSM-5, or ICD-10 diagnostic criteria. Psychological Medicine. 2018 Nov 29;49(08):1237–49. 8. Meier M, Jansen K, Vertgewall H, Claes L. The Lifetime Prevalence of Non-Suicidal Self-Injury in Children and Adolescents With Eating Disorders-A Systematic Review and Meta-Analysis. Eur Eat Disord Rev. 2025 May;33(3):511-524. doi: 10.1002/erv.3158. Epub 2024 Nov 30. PMID: 39614701; PMCID: PMC11965553. 9. Pompili M, Mancinelli I, Girardi P, Ruberto A, Tatarelli R. Suicide in anorexia nervosa: A meta-analysis. International Journal of Eating Disorders. 2004;36(1):99–103. 10. Preti A, Rocchi MBL, Sisti D, Camboni MV, Miotto P. A comprehensive meta-analysis of the risk of suicide in eating disorders. Acta Psychiatrica Scandinavica. 2010 Nov 24;124(1):6–17. PMID: 21092024. 11. Sesboüé S, Grandclerc S, Moro MR, Godart N, Blanchet C. Non-suicidal self-injury and anorexia nervosa: A systematic scoping review. Encephale. 2025 Feb 14:S0013-7006(25)00011-9. doi: 10.1016/j.encep.2024.11.017. Epub ahead of print. PMID: 39955240. 12. Sohn MN, Dimitropoulos G, Ramirez A, McPherson C, Anderson A, Munir A, Patten SB, McGirr A, Devoe DJ. Non-suicidal self-injury, suicidal thoughts and behaviors in individuals with an eating disorder relative to healthy and psychiatric controls: A systematic review and meta-analysis. Int J Eat Disord. 2023 Mar;56(3):501-515. doi: 10.1002/eat.23880. Epub 2023 Jan 16. PMID: 36647184. 13. Smith AR, Velkoff EA, Ribeiro JD, Franklin J. Are eating disorders and related symptoms risk factors for suicidal thoughts and behaviors? A meta-analysis. Suicide Life Threat Behav . 2019;49(1):221–39. Available from: http://dx.doi.org/10.1111/sltb.12427. 14. Zelkowitz et al. *Suicide and Life-Threatening Behavior* 2018. **Self-Criticism as a Transdiagnostic Process in Nonsuicidal Self-Injury and Disordered Eating: Systematic Review and Meta-Analysis** [Self‐Criticism as a Transdiagnostic Process in Nonsuicidal Self‐Injury and Disordered Eating: Systematic Review and Meta-Analysis - Zelkowitz - 2019 - Suicide and Life-Threatening Behavior - Wiley Online Library](https:/onlinelibrary.wiley.com/doi/10.1111/sltb.12436).

## Appendix

**Table 5:** JBI analysis of the included systematic reviews (conducted by reviewer RW)

	Amiri, 2023	Conti, 2017	Cucchi, 2016	Eisenman, 2025	Goldstein, 2019	Kirkpatrick, 2024	Mandelli, 2018	Meier, 2024	Pompili, 2004	Preti, 2010	Sesboüé, 2025	Smith, 2018	Sohn, 2023	Zelkowitz, 2018
Is the review question clearly and explicitly stated?	Yes	Yes	Yes	Yes	Yes	Yes	Yes	Yes	No	Yes	Yes	Yes	Yes	Yes
Were the inclusion criteria appropriate for the review question?	Yes	Yes	Yes	Yes	Yes	Yes	Yes	Yes	Unclear	Unclear	Yes	Yes	Yes	No
Was the search strategy appropriate?	Yes	Yes	Yes	Yes	No	Yes	Yes	Yes	No	Yes	Yes	Yes	Yes	Yes
Were the sources and resources used to search for studies adequate?	Yes	Yes	Yes	Yes	Yes	Yes	Unclear	Yes	No	No	Yes	Yes	Yes	Yes
Were the criteria for appraising studies appropriate?	Yes	No	No	Yes	No	Yes	Yes	No	No	No	No	No	Yes	No
Was critical appraisal conducted by two or more reviewers independently?	No	No	No	Yes	No	Yes	Yes	No	No	No	No	No	Yes	No
Were there methods to minimize errors in data extraction?	Yes	No	Yes	Yes	No	Yes	No	Yes	No	No	No	No	Yes	Yes
Were the methods used to combine studies appropriate?	Yes	Yes	Yes	Yes	Yes	Yes	Yes	Yes	No	Yes	Yes	Yes	Yes	Yes
Was the likelihood of publication bias assessed?	No	No	No	No	No	No	Yes	No	No	Yes	No	No	No	Yes
Were recommendations for policy and/or practice supported by the reported data?	Yes	Yes	Yes	Yes	Yes	Yes	Yes	Yes	N/a	N/a	Yes	Yes	Yes	Yes
Were the specific directives for new research appropriate?	Yes	No	Yes	Yes	Yes	Yes	Yes	Yes	No	Yes	Yes	No	Yes	Yes
Score out of 11	9	6	8	10	6	10	9	8	0	4	7	6	10	8
Percentage	81	54	72	91	54	91	81	72	0	36	63	55	91	72

## Abbreviations

AMSTAR 2: A MeaSurement Tool to Assess systematic Reviews 2
AN: Anorexia Nervosa
AN-BP: Anorexia Nervosa, Biniging Purging subtype
AN-R: Anorexia Nervosa, Restrictive subtype
ARFID: Avoidant-Restrictive Food Intake Disorder
BED: Binge Eating disorder
BN: Bulimia Nervosa
C-SSRS: Colombia – Suicide Severity Rating Scale
DSM V: Diagnostic and Statistical Manual of Mental Disorders V
EDNOS: Eating disorders not specified
ICD 11: International Classification of Disease 11
IMV: Integrated motivational-volitional model of suicidal behaviour
IPTS: Interpersonal Psychological Theory of suicidal behaviour
JBI: Joanna Briggs Institute
NSI: Non-suicidal self-injury
OSFED: Other unspecified eating disorders
ROBIS: Risk of Bias Assessment
